# Taming hyperparameter tuning in continuous normalizing flows using the JKO scheme

**DOI:** 10.1038/s41598-023-31521-y

**Published:** 2023-03-18

**Authors:** Alexander Vidal, Samy Wu Fung, Luis Tenorio, Stanley Osher, Levon Nurbekyan

**Affiliations:** 1 Department of Applied Mathematics and Statistics, Colorado School of Mines, Golden, USA; 2 Department of Applied Mathematics and Statistics, Department of Computer Science, Colorado School of Mines, Golden, USA; 3 Department of Mathematics, University of California, Los Angeles, USA

**Keywords:** Computational science, Applied mathematics

## Abstract

A normalizing flow (NF) is a mapping that transforms a chosen probability distribution to a normal distribution. Such flows are a common technique used for data generation and density estimation in machine learning and data science. The density estimate obtained with a NF requires a change of variables formula that involves the computation of the Jacobian determinant of the NF transformation. In order to tractably compute this determinant, continuous normalizing flows (CNF) estimate the mapping and its Jacobian determinant using a neural ODE. Optimal transport (OT) theory has been successfully used to assist in finding CNFs by formulating them as OT problems with a soft penalty for enforcing the standard normal distribution as a target measure. A drawback of OT-based CNFs is the addition of a hyperparameter, $\alpha$, that controls the strength of the soft penalty and requires significant tuning. We present JKO-Flow, an algorithm to solve OT-based CNF without the need of tuning $\alpha$. This is achieved by integrating the OT CNF framework into a Wasserstein gradient flow framework, also known as the JKO scheme. Instead of tuning $\alpha$, we repeatedly solve the optimization problem for a fixed $\alpha$ effectively performing a JKO update with a time-step $\alpha$. Hence we obtain a ”divide and conquer” algorithm by repeatedly solving simpler problems instead of solving a potentially harder problem with large $\alpha$.

## Introduction

A normalizing flow (NF) is a type of generative modeling technique that has shown great promise in applications arising in physics^1–3^ as a general framework to construct probability densities for continuous random variables in high-dimensional spaces^4–6^. An NF provides a ${\mathcal {C}}^1$-diffeomorphism *f* (i.e., a normalizing transformation) that transforms the density $\rho _0$ of an initial distribution $P_0$ to the density $\rho _1$ of the standard multivariate normal distribution $P_1$—hence the term ”normalizing”. Given such mapping *f*, the density $\rho _0$ can be recovered from the Gaussian density via the change of variables formula,

1 $$\begin{aligned} \log \rho _0(x) = \log \rho _1\left( f(x)\right) + \log |\det J_f(x)|, \end{aligned}$$

where $J_f \in \mathbb {R}^{d \times d}$ is the Jacobian of *f*. Moreover, one can obtain samples with density $\rho _0$ by pushing forward Gaussian samples via $f^{-1}$.

### Remark 1

Throughout the paper we slightly abuse notation, using the same notation for probability distributions and their density functions. Additionally, given a probability distribution $P_0$ on $\mathbb {R}^d$ and a measurable mapping $f:\mathbb {R}^d \rightarrow \mathbb {R}^d$, we define the pushforward distribution of $P_0$ through *f* as $(f\sharp P_0)(B)=P_0(f^{-1}(B))$ for all Borel measurable $B\subseteq \mathbb {R}^d$^7,8^.

There are two classes of normalizing flows: finite and continuous. A finite flow is defined as a composition of a finite number of $\mathcal {C}^1$-diffeomorphisms: $f = f_1 \circ f_2 \circ \cdots \circ f_n$. To make finite flows computationally tractable, each $f_i$ is chosen to have some regularity properties such as a Jacobian with a tractable determinant; for example, $J_{f_i}$ may have a triangular structure^9–11^.

On the other hand, continuous normalizing flows (CNFs) estimate *f* using a neural ODE of the form^12^:

2 $$\begin{aligned} \partial _t z(x,t) = v_\theta (z(x,t),t), \qquad z(x,0) = x, \qquad 0 \le t \le T, \end{aligned}$$

where $\theta$ are the parameters of the neural ODE. In this case, *f* is defined as $f(x)= z(x,T)$ (for simplicity, we remove the dependence of *z* on $\theta$).

One of the main advantages of CNFs is that we can tractably estimate the log-determinant of the Jacobian using Jacobi’s identity, which is commonly used in fluid mechanics (see, e.g.,^8^, p. 114):

3 $$\begin{aligned} \begin{aligned} \partial _t \log |\det \nabla _x z(x,t)|&= \nabla _z \cdot v_\theta (z(x,t),t) = {\text {trace}}\left( \nabla _z v_\theta (z(x,t),t)\right) . \end{aligned} \end{aligned}$$

This is computationally appealing as one can replace the expensive determinant calculation by a more tractable trace computation of $\nabla _z v_\theta (z(x,t),t)$. Importantly, no restrictions on $\nabla _z v_\theta (z(x,t),t)$ (e.g., diagonal or triangular structure) are needed; thus, these Jacobians are also referred to as “free-form Jacobians”^13^.

The goal in training a CNF is to find parameters, $\theta$, such that $f=z(\cdot ,T)$ leads to a good approximation of $\rho _1$ or, assuming *f* is invertible, the pushforward of $\rho _1$ through $f^{-1}$ is a good approximation of $\rho _0$^5,6,10,13^. Indeed, let $\widehat{\rho }_0$ be this pushforward density obtained with a CNF *f*; that is, $\widehat{\rho }_0=f^{-1}\sharp \rho _1$. We then minimize the Kullback-Leibler (KL) divergence from $\widehat{\rho }_0$ to $\rho _0$ given by

$$\begin{aligned} \min _{\theta }~ \mathbb {E}_{x \sim \rho _0} \log ( \rho _0(x)/\widehat{\rho }_0(x))= \min _{\theta }~ \mathbb {E}_{x \sim \rho _0}\left[ \log \rho _0(x) - \log \rho _1(z(x,T)) - \ell (x,T)\right] , \end{aligned}$$

where $\ell (x,T) = \log | \det \nabla z(x,T) |$. Dropping the $\theta$-independent term $\log \rho _0$ and using Eqs. (2) and (3), this previous optimization problem reduces to the minimization problem

4 $$\begin{aligned} \min _{\theta } \;\; \mathbb {E}_{x \sim \rho _0}\; C(x,T), \quad C(x,T):= -\log \rho _1(z(x,T)) - \ell (x,T) \end{aligned}$$

subject to ODE constraints

5 $$\begin{aligned} \partial _t \begin{bmatrix} z(x,t) \\ \ell (x,t) \end{bmatrix} = \begin{bmatrix} v_\theta (z(x,t), t) \\ {\text {trace}}\left( \nabla _z v_\theta (z(x,t),t)\right) \end{bmatrix}, \qquad \begin{bmatrix} z(x,0) \\ \ell (x,0) \end{bmatrix} = \begin{bmatrix} x \\ 0 \end{bmatrix}. \end{aligned}$$

The ODE Eq. (5) might be stiff for certain values of $\theta$, leading to extremely long computation times. Indeed, the dependence of *v* on $\theta$ is highly nonlinear and might generate vector fields that lead to highly oscillatory trajectories with complex geometry.

Some recent work leverages optimal transport theory to find the CNF^14,15^. In particular, a kinetic energy regularization term (among others) is added to the loss to “encourage straight trajectories” *z*(*x*, *t*). That is, the flow is trained by solving the following minimization instead of Eq. (4):

6 $$\begin{aligned} \begin{aligned} \min _{\theta } \;\; \mathbb {E}_{x \sim \rho _0} \; \int _0^T \frac{1}{2} \Vert v_\theta (z(x,t),t)\Vert ^2 dt + \alpha C(x,T) \end{aligned} \end{aligned}$$

subject to Eq. (5). The key insight in^14,15^ is that Eq. (4) is an example of a degenerate OT problem with a soft terminal penalty and without a transportation cost. The first term in the objective function in Eq. (6) given by the time integral is the transportation cost, whereas $\alpha$ is a hyperparameter that balances the soft penalty and the transportation cost. Including this cost makes the problem well-posed by forcing the solution to be unique^16^. Additionally, it enforces straight trajectories so that Eq. (5) is not stiff. Indeed^14,15^ empirically demonstrate that including optimal transport theory leads to faster and more stable training of CNFs. Intuitively, we minimize the KL divergence *and* the arclength of the trajectories.

Although including optimal transport theory into CNFs has been very successful^14,15,17,18^, there are two key challenges that render them difficult to train. First, estimating the log-determinant in Eq. (4) via the trace in Eq. (5) is still computationally taxing and commonly used methods rely on stochastic approximations^13,14^, which add extra error. Second, including the kinetic energy regularization requires tuning of the hyperparameter $\alpha$. Indeed, if $\alpha$ is chosen too small in Eq. (6), then the kinetic regularization term dominates the training process, and the optimal solution consists of not moving, i.e., $f(x) = x$. On the other hand, if $\alpha$ is chosen too large, we return to the original setting where the problem is ill-posed, i.e., there are infinitely many solutions. Finally, finding an ”optimal” $\alpha$ is problem dependent and requires tuning on a case-by-case basis.

### Our contribution

We present JKO-Flow, an optimal transport-based algorithm for training CNFs without the need to tune the hyperparameter $\alpha$ in Eq. (6). Our approach also leverages fast numerical methods for exact trace estimation from the recently developed optimal transport flow (OT-Flow)^15,19^.

The key idea is to integrate the OT-Flow approach into a Wasserstein gradient flow framework, also known as the Jordan, Kinderlehrer, and Otto (JKO) scheme^20^. Rather than tuning the hyperparameter $\alpha$ (commonly done using a grid search), the idea is to simply pick any $\alpha$ and solve a sequence of ”easier” OT problems that gradually approach the target distribution. Each solve is precisely a gradient descent in the space of distributions, a Wasserstein gradient descent, and the scheme provably converges to the desired distribution for all $\alpha >0$^21^. Our experiments show that our proposed approach is effective in generating higher quality samples (and density estimates) and also allows us to reduce the number of parameters required to estimate the desired flow.

Our strategy is reminiscent of debiasing techniques commonly used in inverse problems. Indeed, the transportation cost that serves as a regularizer in Eq. (6) introduces a bias—the smaller $\alpha$ the more bias is introduced (see, e.g.,^22^), so good choices of $\alpha$ tend to be larger. One way to remove the bias and avoid the need to tune the regularization parameter is to perform a sequence of Bregman iterations^23,24^, also known as nonlinear proximal steps. Hence our approach reduces to debiasing via Bregman or proximal steps in the Wasserstein space. In the context of CNF training, Bregman iterations are advantageous due to the flexibility of the choice for $\alpha$. Indeed, the resulting loss function is non-convex and its optimization tends to get harder for large $\alpha$. Thus, instead of solving one harder problem we solve several “easier” problems.

## Optimal transport background and connections to CNFs

Denote by $\mathcal {P}_2(\mathbb {R}^d)$ the space of Borel probability measures on $\mathbb {R}^d$ with finite second-order moments, and let $\rho _0,\rho _1 \in \mathcal {P}_2(\mathbb {R}^d)$. The quadratic optimal transportation (OT) problem (which also defines the Wasserstain metric $W_2$) is then formulated as

7 $$\begin{aligned} W_2^2(\rho _0,\rho _1)=\inf _{\pi \in \Gamma (\rho _0,\rho _1)} \int _{\mathbb {R}^{2d}} \Vert x-y\Vert ^2 d\pi (x,y), \end{aligned}$$

where $\Gamma (\rho _0,\rho _1)$ is the set of probability measures $\pi \in \mathcal {P}(\mathbb {R}^{2d})$ with fixed *x* and *y*-marginal distributions $\rho _0$ and $\rho _1$, respectively. Hence the cost of transporting a unit mass from *x* to *y* is $\Vert x-y\Vert ^2$, and one attempts to transport $\rho _0$ to $\rho _1$ as cheaply as possible. In Eq. (7), $\pi$ represents a *transportation plan*, and $\pi (x,y)$ is the mass being transported from *x* to *y*. One can prove that $(\mathcal {P}_2(\mathbb {R}^d),W_2)$ is a complete separable metric space^8^. OT has recently become a very active research area in PDE, geometry, functional inequalities, economics, data science and elsewhere partly due to equipping the space of probability measures with a (Riemannian) metric^8,16,25,26^.

As observed in prior works, there are many similarities between OT and NFs^14,15,18,27^. This connection becomes more transparent when considering the dynamic formulation of Eq. (7). More precisely, the Benamou-Brenier formulation of the OT problem is given by^28^:

8 $$\begin{aligned} \begin{aligned} \frac{T}{2} W_2^2(\rho _0, \rho _1) = \inf _{v, \rho } \;\;&\int _0^T \int _{\mathbb {R}^d} \frac{1}{2}\Vert v(x,t)\Vert _2^2 \rho (x,t) dx dt \\ \text{ s.t. } \;\;&\partial _t \rho (x,t) + \nabla \cdot (\rho (x,t) v(x,t)) = 0 \\&\rho (x,0) = \rho _0(x), \;\; \rho (x,T) = \rho _1(x). \end{aligned} \end{aligned}$$

Hence, the OT problem can be formulated as a problem of flowing $\rho _0$ to $\rho _1$ with a velocity field *v* that achieves minimal kinetic energy. The optimal velocity field *v* has several appealing properties. First, particles induced by the optimal flow *v* travel in straight lines. Second, particles travel with constant speed. Moreover, under suitable conditions on $\rho _0$ and $\rho _1$, the optimal velocity field is unique^8^.

Given a velocity field *v*, denote by *z*(*x*, *t*) the solution of the ODE

$$\begin{aligned} \partial _t z(x,t) = v(z(x,t),t), \qquad z(x,0) = x, \qquad 0 \le t \le T. \end{aligned}$$

Then, under suitable regularity conditions, we have that the solution of the continuity equation is given by $\rho (\cdot ,t)=z(\cdot ,t)\sharp \rho _0$. Thus the optimization problem in Eq. (8) can be written as

9 $$\begin{aligned} \begin{aligned} \inf _{v} \;\;&\int _0^T \int _{\mathbb {R}^d} \frac{1}{2}\Vert v(z(x,t),t)\Vert _2^2 \rho _0(x) dx dt \\ \text{ s.t. } \;\;&\partial _t z(x,t) = v(z(x,t),t),~ z(x,0) = x,~ z(\cdot ,T)\sharp \rho _0=\rho _1. \end{aligned} \end{aligned}$$

This previous problem is very similar to (4) with the following differences:

- the objective function in Eq. (4) does not have the kinetic energy of trajectories,
- the terminal constraint is imposed as a soft constraint in Eq. (4) and as a hard constraint in Eq. (9), and
- *v* in Eq. (4) is $\theta$-dependent, whereas the formulation in Eq. (9) is in the non-parametric regime.

So the NF defined by Eq. (4) can be thought of as an approximation to a degenerate transportation problem that lacks transportation cost. Based on this insight one can regularize Eq.  (4) by adding the transportation cost and arrive at Eq. (6) or some closely related version of it^14,15,18,27^. It has been observed that the transportation cost (kinetic energy) regularization significantly improves the training of NFs.

## JKO-flow: Wasserstein gradient flows for CNFs

While the OT-based formulation of CNFs in Eq. (6) has been found successful in some applications^14,15,18,27^, a key difficulty arises in choosing how to balance the kinetic energy term and the KL-divergence, i.e., on selecting $\alpha$. This difficulty is typical in problems where the constraints are imposed in a soft fashion. Standard training of CNFs typically involves tuning for a “large but hopefully stable enough” step size $\alpha$ so that the KL divergence term is sufficiently small after training. To this end, we propose an approach that avoids the need to tune $\alpha$ by using the fact that the solution to Eq. (6) is an approximation to a backward Euler (or proximal point) algorithm when discretizing the Wasserstein gradient flow using the Jordan–Kinderlehrer–Otto (JKO) scheme^20^.

The seminal work in^20^ provides a gradient flow structure of the Fokker–Planck equation using an implicit time discretization. That is, given $\alpha > 0$, density at $k{\text {th}}$ iteration, $\rho ^{(k)}$, and terminal density $\rho _1$, one finds

10 $$\begin{aligned} \begin{aligned} \rho ^{(k+1)} =&\mathop {\mathrm {arg\,min}}\limits _{\rho \in \mathcal {P}_2(\mathbb {R}^d)} \; \frac{1}{2\alpha } W_2^2(\rho , \rho ^{(k)}) + KL(\rho ||\rho _1)\\ =&\mathop {\mathrm {arg\,min}}\limits _{v} \; \frac{1}{\alpha }\int _0^1 \int _{\mathbb {R}^d} \frac{1}{2}\Vert v(z(x,t),t)\Vert _2^2 \rho _0(x) dx dt + KL(z(\cdot ,1)\sharp \rho ^{(k)}||\rho _1)\\ \text{ s.t. } \;\;&\partial _t z(x,t) = v(z(x,t),t),~ z(x,0) = x \end{aligned} \end{aligned}$$

for $k=0,1,\ldots$, and $\rho ^{(0)} = \rho _0$. Here, $\alpha$ takes the role of a step size when applying a proximal point method to the KL divergence using the Wasserstein-2 metric, and $\{\rho ^{(k)}\}$ provably converges to $\rho _1$^20,21^. Hence, repeatedly solving Eq. (9) with the KL penalty acting as a soft constraint yields an arbitrarily accurate approximation of $\rho _1$. In the parametric regime each iteration takes the form

11 $$\begin{aligned} \mathop {\mathrm {arg\,min}}\limits _{\theta } \;\; \mathbb {E}_{x \sim \rho ^{(k)}} \; \int _0^T \frac{1}{2} \Vert v_\theta (x,t)\Vert ^2 dt + \alpha C(x,T) \quad \text {subject to} \quad 5. \end{aligned}$$

Thus we solve a sequence of problems Eq. (6), where the initial density of the current subproblem is given by the pushforward of the density generated in the previous subproblem.

Importantly, our proposed approach *does not require tuning* $\alpha$. Instead, we solve a sequence of subproblems that is guaranteed to converge to $\rho _1$^20^ prior to the neural network parameterization; see Algorithm 1. Indeed, since the traditional approach is equivalent to JKO-Flow with one iteration, JKO-Flow is generally more computationally expensive. But we crucially note that in the traditional single-shot setting, tuning $\alpha$ may require training the model many times as well. JKO-Flow provides a way to automate hyperparameter tuning of $\alpha$. In our experiments, we observe that ten iterations of JKO-Flow leads to good results for a high-dimensional physics problem, see “Numerical experiments”. While our proposed methodology can be used in tandem with any algorithm used to solve Eq. (11), an important numerical aspect in our approach is to leverage fast computational methods that use *exact* trace estimation in Eq. (5); this approach is called OT-Flow^15^. Consequently, we avoid the use of stochastic approximation methods for the trace, e.g., Hutchinson’s estimator^22,29,30^, as is typically done in CNF methods^13,14^. A surprising result of our proposed method is that it empirically shows improved performance even with fewer number of parameters (see Fig. 3).
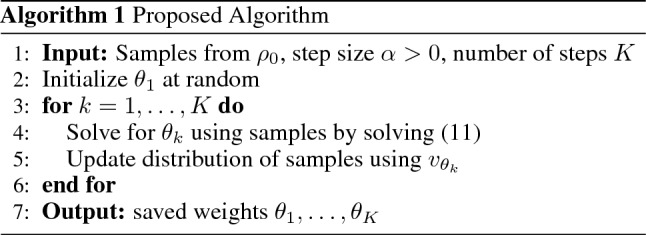


## Related works

### Density estimation

Multivariate density estimation is a fundamental problem in statistics^31,32^, High Energy Physics (HEP)^33^ and in other fields of science dealing with multivariate data. For instance, particle physicists in HEP study possible distributions from a set of high energy data. Another application of density estimation is in confidence level calculations of particles in Higgs searches at Large Electron Positron Colliders (LEP)^34^ and discriminant methods used in the search for new particles^33^. One of the main advantages of NFs over other generative models is that they provide density estimates of probability distributions using Eq. (1). That is, we do not need to apply a separate density estimation technique after generating samples from a distribution, e.g., as in GANs^35^.

### Finite flows

Finite normalizing flows^4–6,36^ use a composition of discrete transformations, where specific architectures are chosen to allow for efficient inverse and Jacobian determinant computations. NICE^37^, RealNVP^38^, IAF^39^, and MAF^10^ use either autoregressive or coupling flows where the Jacobian is triangular, so the Jacobian determinant can be tractably computed. GLOW^40^ expands upon RealNVP by introducing an additional invertible convolution step. These flows are based on either coupling layers or autoregressive transformations, whose tractable invertibility allows for density evaluation and generative sampling. Neural Spline Flows^41^ use splines instead of the coupling layers used in GLOW and RealNVP. Using monotonic neural networks, NAF^42^ require positivity of the weights, which UMNN^43^ circumvent this requirement by parameterizing the Jacobian and then integrating numerically. A recent work, called Normalizing Field Flows (NFFs)^44^, generalizes NFs to include learning random fields from scattered measurements; in particular, NFFs can be used to solve data-driven forward, inverse, and mixed forward/inverse stochastic partial differential equations.

### Continuous and optimal transport-based flows

Modeling flows with differential equations is a natural and commonly used method^27,45–50^. In particular, CNFs model their flow via a neural ordinary differential equation^12,13,51^. Among the most well-known CNFs are FFJORD^13^, which estimates the determinant of the Jacobian by accumulating its trace along the trajectories, and the trace is estimated using Hutchinson’s estimator^22,29,30^. To promote straight trajectories, RNODE^14^ regularizes FFJORD with a transport cost $L(\varvec{x},T)$. RNODE also includes the Frobenius norm of the Jacobian $\Vert \nabla \textbf{v}\Vert _F^2$ to stabilize training. The trace and the Frobenius norm are estimated using a stochastic estimator and report speedup by a factor of 2.8.

Monge-Ampère Flows^18^ and Potential Flow Generators^17^ similarly draw from OT theory but parameterize a potential function instead of the dynamics directly. OT is also used in other generative models^52–57^. OT-Flow^15^ is based on a discretize-then-optimize approach^58^ that also parameterizes the potential function. To evaluate the KL divergence, OT-Flow estimates the density using an *exact* trace computation following the work of^19^.

### Wasserstein gradient flows

Our proposed method is most closely related to^59^, which also employs a JKO-based scheme to perform generative modeling. But a key difference is that^59^ reformulates the KL-divergence as an optimization over difference of expectations (see^59^, Prop. 3.1); this makes their approach akin to GANs, where the density cannot be obtained without using a separate density estimation technique. Our proposed method is also closely related to methods that use input-convex CNNs^60–62^. Reference^62^ focuses on the special case with KL divergence as objective function.^60^ solve a sequence of subproblems different from the fluid flow formulation presented in Eq. (11). They also require an end-to-end training scheme that backpropagates to the initial distribution; this can become a computational burden when the number of time discretizations is large. Reference^61^ utilizes a JKO-based scheme to approximate a population dynamics given an observed trajectory and focus on applications in computational biology. Other related works include natural gradient methods^63^ and implicit schemes based on the Wasserstein-1 distance^64^.

## Numerical experiments

We demonstrate the effectiveness of our proposed JKO-Flow on a series of synthetic and real-world datasets. As previously mentioned, we compute each update in Eq. (10) by solving Eq. (6) using the OT-Flow solver^15^, which leverages fast and exact trace computations. We also use the same architecture provided in^15^. Henceforth, we shall also call the traditional CNF approach the “single-shot” approach. We also clarify that $\alpha$ in our experiments refers to the parameter in Eq. (6).

### Maximum mean discrepancy metric (MMD)

Our density estimation problem requires approximating a density $\rho _0$ by finding a transformation *f* such that $f^{-1}\sharp \rho _1$ has density $\widehat{\rho }_0$ close to $\rho _0$, where $\rho _1$ is the standard multivariate Gaussian. However, $\rho _0$ is not known in real-world density estimation scenarios, such as in physics applications, all we have are samples $X = \{x_i\}_{i=1}^n$ from the unknown distribution. Consequently, we use the observed samples *X* and samples $\widehat{X}=\{\widehat{x}_j\}_{j=1}^m$, $\widehat{x}_j=f^{-1}(q_j)$, generated by the CNF and samples $Q=\{q_j\}_{j=1}^m$ from $\rho _1$ to determine if their corresponding distributions are close in some sense. To measure the discrepancy we use a particular integral probability metric^65–67^ known as maximum mean discrepancy (MMD) defined as follows^68^: let *x* and *y* be random vectors in $\mathbb {R}^d$ with distributions $\mu _x$ and $\mu _y$, respectively, and let $\mathcal {H}$ be a reproducing kernel Hilbert space (RKHS) of functions on $\mathbb {R}^d$ with Gaussian kernel (see^69^ for an introduction o RKHS’s)

12 $$\begin{aligned} k(x_i, x_j) = \exp {\left( -\frac{1}{2}\Vert x_i - x_j\Vert ^2\right) }. \end{aligned}$$

Then the MMD of $\mu _x$ and $\mu _y$ is given by

$$\begin{aligned} \textrm{MMD}_{\mathcal {H}}(\mu _x,\mu _y) = \sup _{\Vert f\Vert _\mathcal {H}\le 1}\,|\,\mathbb {E}\,f(x) - \mathbb {E}\,f(y)\,|. \end{aligned}$$

It can be shown that $\textrm{MMD}_\mathcal {H}$ defines a metric on the class of probability measures on $\mathbb {R}^d$^68,70^. The squared-MMD can be written in terms of the kernel as follows:

$$\begin{aligned} \textrm{MMD}^2_{\mathcal {H}}(\mu _x,\mu _y) = \mathbb {E}\,k(x,x^\prime ) + \mathbb {E}\,k(y,y^\prime ) -2\,\mathbb {E}\,k(x,y), \end{aligned}$$

where $x,x^\prime$ are iid $\mu _x$ independent of $y,y^\prime$ which are iid $\mu _y$. An unbiased estimate of the squared-MMD based on the samples *X* and $\widehat{X}$ defined above is given by^68^:

$$\begin{aligned} \textrm{MMD}^2_\mathcal {H}(X,\widehat{X}) = \frac{1}{n(n-1)}\sum _{i\ne j} k(x_i,x_j) + \frac{1}{m(m-1)}\sum _{k\ne \ell } k(\widehat{x}_k,\widehat{x}_\ell ) -\frac{2}{nm}\sum _{i,\ell } k(x_i,\widehat{x}_\ell ). \end{aligned}$$

Note that the MMD is not used for algorithmic training of the CNF, it is only used to compare the densities $\rho _0$ and $\widehat{\rho }_0$ based on the samples *X* and $\widehat{X}$.

### Synthetic 2D data set

**Figure 1:**
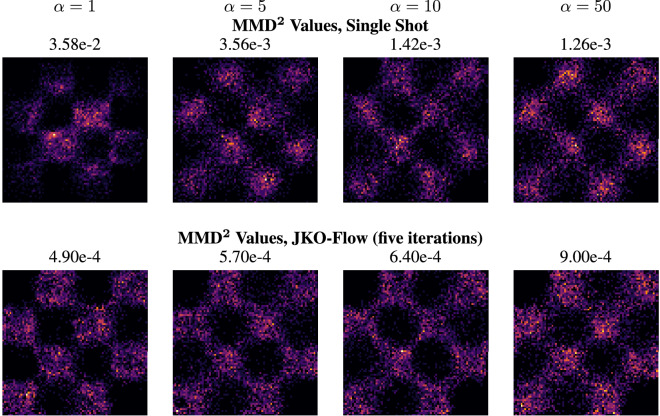
Checkerboard dataset: generated samples of $\widehat{\rho }_0$ using the standard one-shot approach (top row). Generated using our proposed JKO-Flow using five iterations (bottom row). Here, we use $\alpha = 1$, 5, 10, 50. JKO-Flow returns consistent results regardless of the value of $\alpha$.

**Figure 2:**
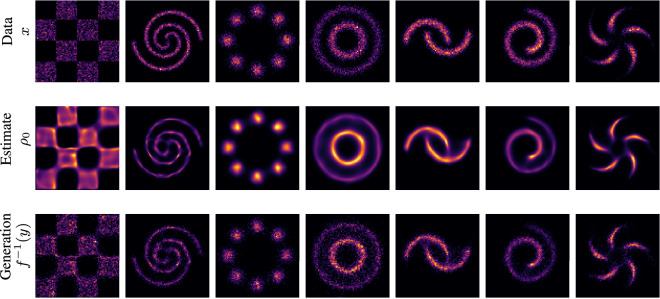
Density estimation on 2D toy problems using five JKO-Flow iterations. Top: samples from the unknown distribution $\rho _0$. Middle: density estimate for $\rho _0$ computed by inverting the flow through the five iterations of JKO-Flow from $\rho _1$ via Eq. (2). Bottom: samples generated by inverse JKO-Flow through five iterations where *y* has density $\rho _1$.

We begin by testing our method on seven two-dimensional (2D) benchmark datasets for density estimation algorithms commonly used in machine learning^13,43^; see Fig. 2. We generate results with JKO-Flow for different values of $\alpha$ and for different number of iterations. We use $\alpha = 1$, 5, 10, and 50, and for each $\alpha$ we use the single shot approach $k=1$ and JKO-Flow with $k=5$ iterations from Eq. (10). Note that in CNFs, we are interested in estimating the density (and generating samples) from $\rho _0$; consequently, once we have the optimal weights $\theta ^{(1)}, \theta ^{(2)}, \ldots , \theta ^{(5)}$, we must “flow backwards” starting with samples from the normal distribution $\rho _1$. Figure 1 shows that JKO-Flow outperforms the single shot approach for different values of $\alpha$. In particular, the performance for the single shot approach varies drastically for different values of $\alpha$, with $\alpha =1$ being an order of magnitude higher in MMD than $\alpha =5$. On the other hand, JKO-Flow performs consistently regardless of the value of $\alpha$ for most datasets. There is one exception for the spirals dataset with $\alpha = 1$; this is because only five iterations are used in the JKO scheme and more are needed. When 10 iterations are used instead, we achieve a similar order of accuracy ($2.3e-4$). As previously mentioned, this is expected as JKO-Flow is a proximal point algorithm that converges regardless of the step size $\alpha$. In this case, five JKO-Flow iterations are enough to obtain this consistency. Additional plots and hyperparameter setups for different benchmark datasets with similar performance results are shown in the Supplementary Information. Table 1 summarizes the comparison between the single shot and JKO-Flow on all synthetic 2D datasets for different values of $\alpha$. We also show an illustration of all the datasets, estimated densities, and generated samples with JKO-Flow in Fig. 2.

**Table 1:** Synthetic 2D data: JKO-flow performance for different values of $\alpha$. JKO-flow returns consistent performance for different $\alpha$.

$\alpha$		1	5	10	50
Dataset	Approach	MMD${^2}$			
Checkerboard	Single shot	3.58e−2	3.56e−3	1.42e−3	1.26e−3
	JKO-flow (5 iters)	4.9e−4	5.67e−4	6.40e−4	9.00e−4
2 spirals	Single shot	7.21e−2	2.30e−2	1.84e−2	7.73e−4
	JKO-flow (5 iters)	2.10e−2	4.62e−4	1.02e−4	5.37e−5
Swiss roll	Single shot	4.74e−3	7.33e−4	2.86e−4	7.03e−4
	JKO-flow (5 iters)	5.16e−4	8.3e−5	3.27e−5	6.07e−4
8 Gaussians	Single shot	9.18e−3	2.69e−4	3.94e−4	7.10e−4
	JKO-flow (5 iters)	1.07e−4	4.13e−5	2.67e−4	7.27e−6
Circles	Single shot	9.84e−3	2.24e−4	6.51e−4	1.04e−4
	JKO-flow (5 iters)	9.49e−4	9.97e−6	2.38e−5	9.28e−5
Pinwheel	Single shot	1.18e−2	1.8e−3	1.37e−3	2.2e−5
	JKO-flow (5 iters)	4.7e−4	2.63e−4	3.84e−4	4.30e−4
Moons	Single shot	1.45e−3	2.05e−3	2.49e−4	2.42e−4
	JKO-flow (5 iters)	1.92e−4	4.65e−5	4.3e−5	1.08e−4

### Varying network size

**Figure 3:**
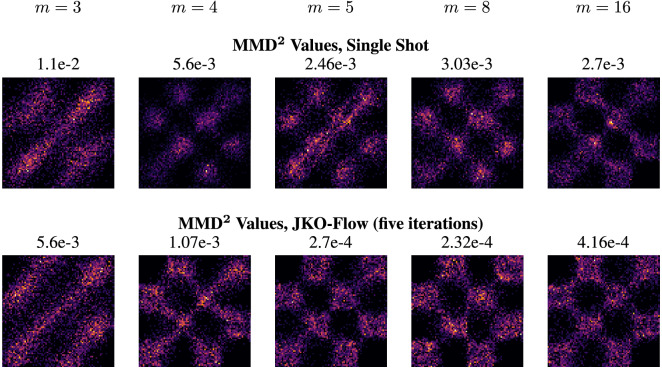
Checkerboard dataset: generated samples of $\widehat{\rho }_0$ using the standard single shot approach (top row). Generated samples using our proposed JKO-Flow using five iterations (bottom row). Here, we fix $\alpha = 5$ and vary the network width $m = 3, 4, 5, 8$, and 16. JKO-Flow performs competitively even with fewer parameters.

In addition to obtaining consistent results for different values of $\alpha$, we also *empirically* observe that JKO-Flow outperforms the single shot approach for different numbers of network parameters, i.e., network size. We illustrate this in Fig. 3. This is also intuitive as we reformulate the problem of finding a single “difficult” optimal transportation problem as a sequence of “smaller and easier” OT problems. In this setup, we vary the width of a two-layer ResNet^71^. In particular, we choose the widths to be $m=3, 4, 5, 8$, and 16. These correspond to 40, 53, 68, 125, and 365 parameters. The hyperparameter $\alpha$ is chosen to be the best performing value for each synthetic dataset. All datasets vary *m* for fixed $\alpha = 5$, except the 2 Spiral dataset, which uses $\alpha =50$; we chose these $\alpha$ values as they performed the best in the fixed *m* experiments. Similar results are also shown for the remaining synthetic datasets in the Supplementary Information. Table 2 summarizes the comparison between the single shot and JKO-Flow on all synthetic 2D datasets.

**Table 2:** Synthetic 2D data: network width comparison for 1 and 5 iterations given a fixed, best performing $\alpha$. JKO-Flow performs better than the single shot approach for different network sizes.

*m*		3	4	5	8	16
Dataset	Approach	MMD${^2}$				
Checkerboard	Single shot	1.10e−2	5.60e−3	2.46e−3	3.03e−3	2.70e−3
	JKO-flow (5 iters)	5.60e−3	1.07e−3	2.7e−4	2.32e−4	4.16e−4
2 spirals	Single shot	5.98e−3	4.54e−3	5.47e−3	1.19e−3	3.96e−3
	JKO-flow (5 iters)	1.42e−3	1.49e−5	6.11e−4	3.93e−5	2.19e−3
Swiss roll	Single shot	8.89e−3	7.71e−3	1.41e−3	1.37e−3	1.52e−3
	JKO-flow (5 iters)	1.49e−3	2.90e−4	6.13e−4	2.29e−4	8.40e−5
8 Gaussians	Single shot	2.20e−3	1.05e−3	1.04e−3	2.3e−4	5.05e−4
	JKO-flow (5 iters)	1.33e−4	9.85e−4	2.40e−5	3.96e−4	1.07e−4
Circles	Single shot	2.06e−3	1.72e−3	1.37e−3	1.69e−3	1.34e−3
	JKO-flow (5 iters)	1.94e−3	3.24e−4	7.71e−4	5.9e−5	1.01e−4
Pinwheel	Single shot	1.10e−2	4.03e−3	2.27e−3	3.80e−3	5.43e−4
	JKO-flow (5 iters)	1.20e−3	8.23e−4	1.60e−3	7.00e−5	2.69e−4
Moons	Single shot	4.98e−3	4.54e−3	5.47e−3	1.2e−3	3.96e−3
	JKO-flow (5 iters)	1.42e−3	1.5e−5	6.11e−4	3.90e−5	2.19e−3

### Density estimation on a physics dataset

**Figure 4:**
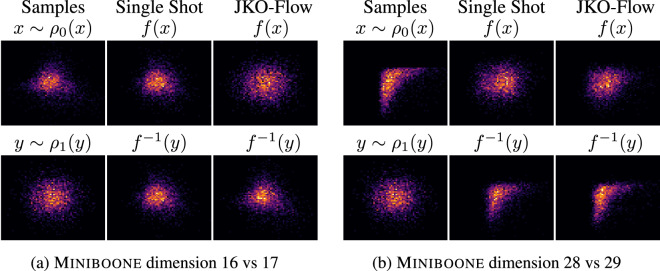
Generated samples for the 43-dimensional Miniboone dataset using the single shot approach and JKO-Flow with 10 iterations. To visualize the dataset, we show 2-dimensional slices. We show the forward flow *f*(*x*) where $x \sim \rho _0$ and the genereated samples $f^{-1}(y)$ where $y \sim \rho _1$.

**Figure 5:**
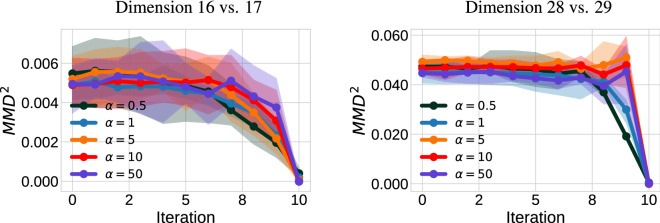
MMD$^2$ per iteration when using JKO-Flow after training. MMD$^2$ values vs. JKO-Flow iteration for 2-dimensional slice (dimensions 16-17 on the left and dimensions 28-29 on the right) of the Miniboone dataset. JKO-Flow achieves same accuracy *regardless* of the value of $\alpha$.

We train JKO-Flow on the 43-dimensional Miniboone dataset which is a high-dimensional, real-world physics dataset used as benchmark for high-dimensional density estimation algorithms in physics^72^. For this physics problem, our method is trained for $\alpha = 0.5, \, 1, \, 5, \, 10,\,50$ and using 10 JKO-Flow iterations. Fig. 4 shows generated samples with JKO-Flow and the standard single-shot approach for $\alpha = 5$. Since Miniboone is a high-dimensional dataset, we follow^15^ and plot two-dimensional slices. JKO-Flow generates better quality samples. Similar experiments for $\alpha =1, 10$, and 50 are shown in the Supplementary Information. Table 3 summarizes the results for all values of $\alpha$. Note that we compute MMD values for all the dimensions as well as 2D slices; this is because we only have limited data (  3000 testing samples) and the 2D slice MMD give a better indication on the improvement of the generated samples. Results show that *the MMD is consistent across all *$\alpha$ *values for JKO-Flow*. We also show the convergence (in MMD$^2$) of the miniboone dataset across each 2D slice in Fig. 5. As expected, smaller step size $\alpha$ values converge slower (see $\alpha = 0.5)$, but all converge to similar accuracy (unlike the single-shot).

**Table 3:** Miniboone: comparison of single shot and JKO-flow for different values of $\alpha$

$\alpha$		0.5	1	5	10	50
Dimensions	Approach	MMD${^2}$				
2d: 16 vs. 17	Single shot	4.62e−3	4.50e−3	5.88e−3	4.89e−3	5.2e−3
	JKO-flow (10 iters)	3.42e−4	2.94e−4	1.27e−4	1.43e−4	1.71e−4
2d: 28 vs. 29	Single shot	4.33e−2	4.60e−2	5.02e−2	4.97e−2	4.74e−2
	JKO-flow (10 iters)	8.02e−5	3.31e−5	4.43e−5	6.33e−5	9.97e−5
Full	Single shot	4.7e−2	4.51e−3	4.75e−3	4.21e−3	4.27e−3
	JKO-flow (10 iters)	4.72e−4	4.72e−4	4.71e−4	4.72e−4	4.72e−4

## Conclusion

We propose a new approach we call JKO-Flow to train OT-regularized CNFs without having to tune the regularization parameter $\alpha$. The key idea is to embed an underlying OT-based CNF solver into a Wasserstein gradient flow framework, also known as the JKO scheme; this approach makes the regularization parameter act as a “time” variable. Thus, instead of tuning $\alpha$, we repeatedly solve proximal updates for a fixed (time variable) $\alpha$. In our setting, we choose OT-Flow^15^, which leverages exact trace estimation for fast CNF training. Our numerical experiments show that JKO-Flow leads to improved performance over the traditional approach. Moreover, *JKO-Flow achieves similar results regardless of the choice of *$\alpha$. We also empirically observe improved performance when varying the size of the neural network. Future work will investigate JKO-Flow on similar problems such as deep learning-based methods for optimal control^73–75^ and mean field games^19,76,77^.

## Supplementary Information

Supplementary Information.

## Data Availability

The datasets used and/or analysed during the current study available from the corresponding author on reasonable request.
