# Supplementary material for: Taming hyperparameter tuning in continuous normalizing flows using the JKO scheme
Source: Sci Rep. 2023 Mar 18;13:4501. doi: 10.1038/s41598-023-31521-y (PMC10024737; doi:10.1038/s41598-023-31521-y)
Supplement: Supplementary file 1 — Supplementary Information. [file 41598_2023_31521_MOESM1_ESM.pdf]

Supplementary Information: Taming Hyperparameter Tuning in Continuous Normalizing Flows Using the JKO Scheme

Alexander Vidal **Samy Wu Fung** **Luis Tenorio** **Stanley Osher** **Levon Nurbekyan**

1 Additional Experiments

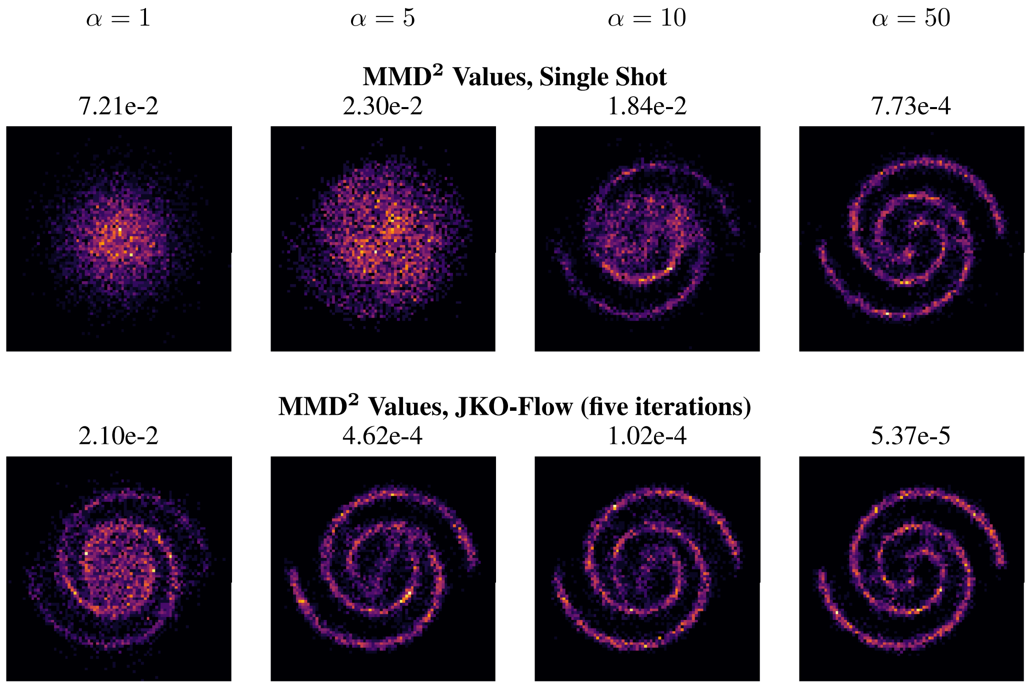

Supplementary Figure 1: 2 Spirals dataset: Generated samples of $\hat{\rho}_0$ using the standard one-shot approach (top row). Generated using our proposed JKO-Flow using five iterations (bottom row). Here, we use $\alpha = 1$, 5, 10, 50. JKO-Flow returns consistent results *regardless of the value of* α.

Preprint. Under review.

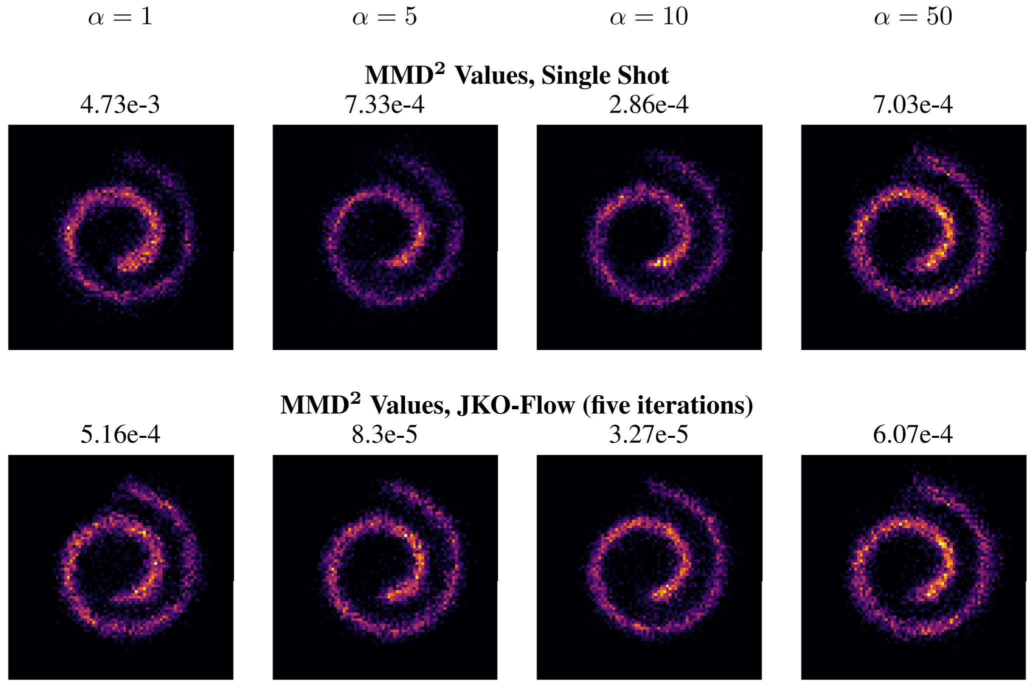

Supplementary Figure 2: Swiss Roll dataset: Generated samples of $\hat{\rho}_0$ using the standard one-shot approach (top row). Generated using our proposed JKO-Flow using five iterations (bottom row). Here, we use $\alpha = 1, 5, 10, 50$. JKO-Flow returns consistent results *regardless of the value of* α.

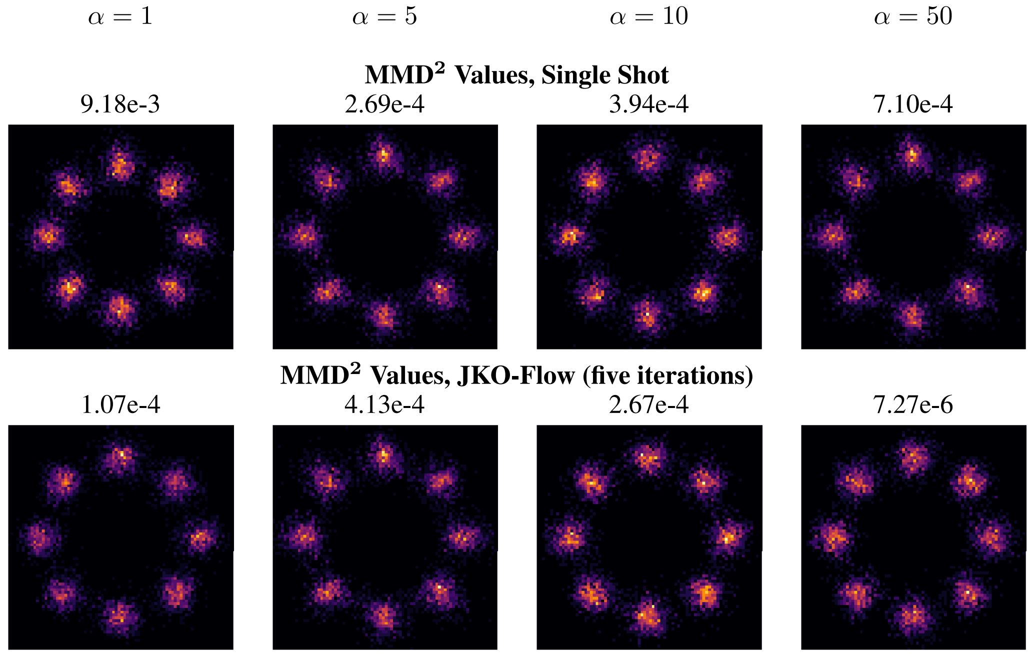

Supplementary Figure 3: 8 Gaussians dataset: Generated samples of $\hat{\rho}_0$ using the standard one-shot approach (top row). Generated using our proposed JKO-Flow using five iterations (bottom row). Here, we use $\alpha = 1, 5, 10, 50$. JKO-Flow returns consistent results *regardless of the value of* α.

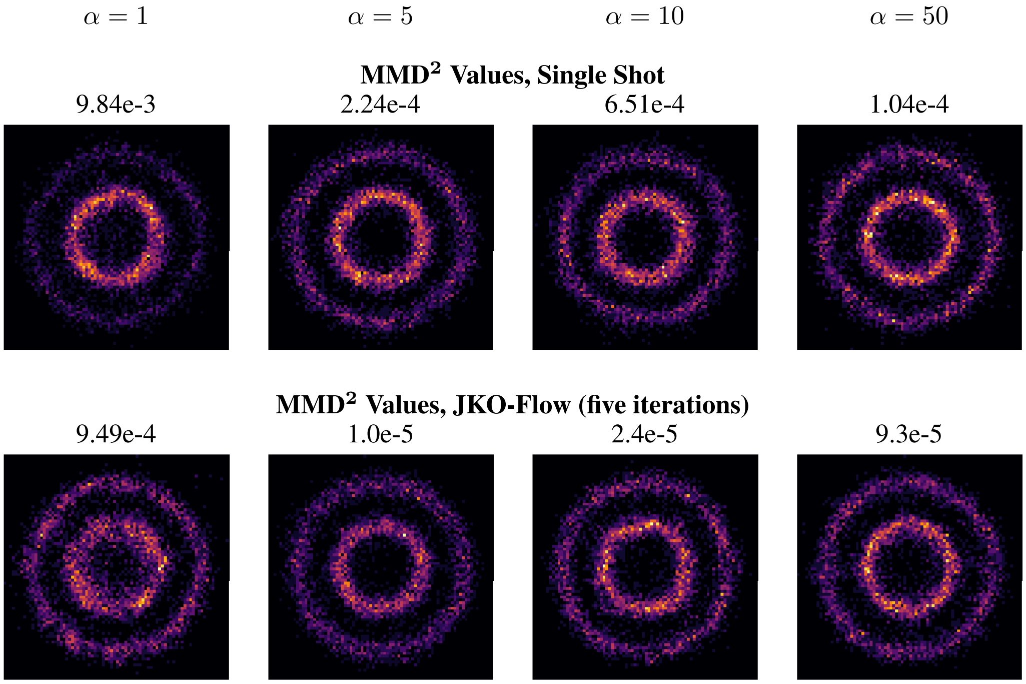

Supplementary Figure 4: Circles dataset: Generated samples of $\hat{\rho}_0$ using the standard one-shot approach (top row). Generated using our proposed JKO-Flow using five iterations (bottom row). Here, we use $\alpha = 1, 5, 10, 50$. JKO-Flow returns consistent results *regardless of the value of* α.

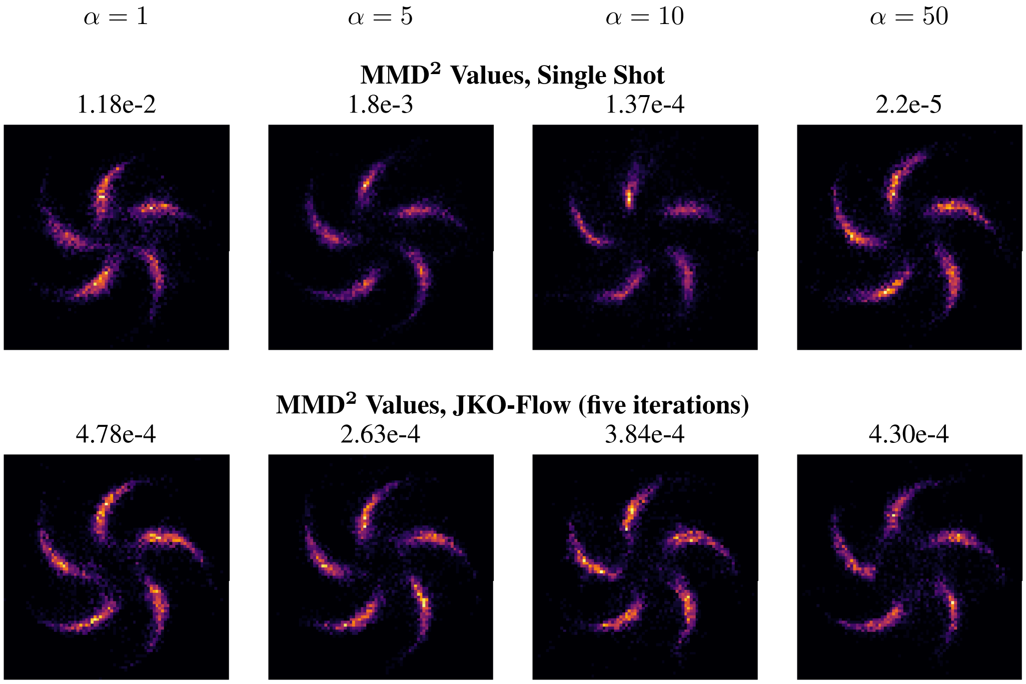

Supplementary Figure 5: Pinwheel dataset: Generated samples of $\hat{\rho}_0$ using the standard one-shot approach (top row). Generated using our proposed JKO-Flow using five iterations (bottom row). Here, we use $\alpha = 1, 5, 10, 50$. JKO-Flow returns consistent results *regardless of the value of* α.

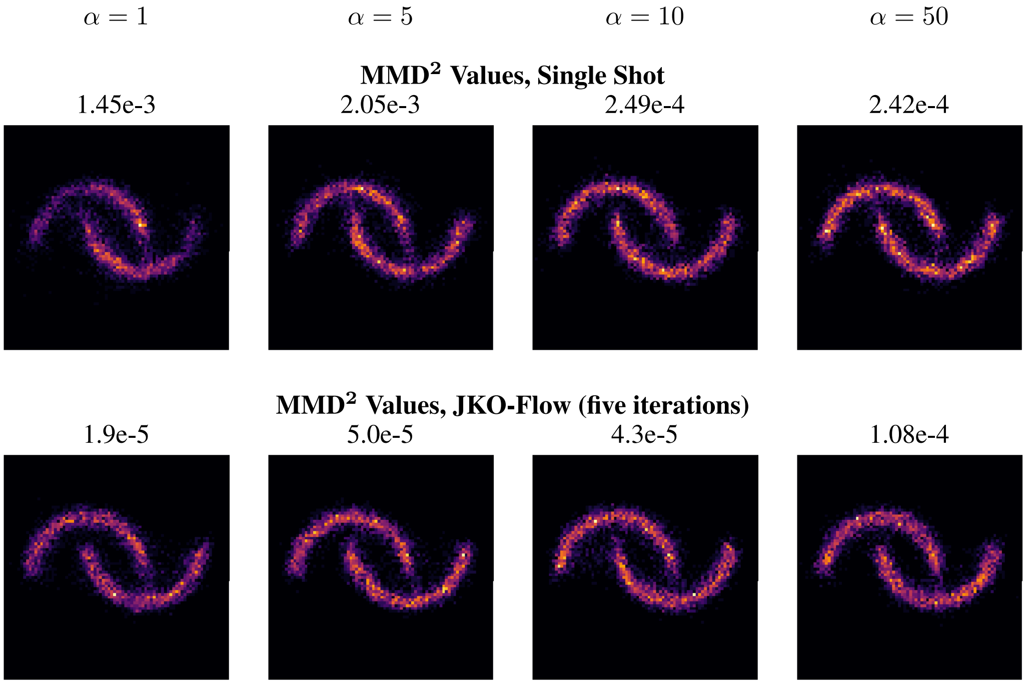

Supplementary Figure 6: Moons dataset: Generated samples of $\hat{\rho}_0$ using the standard one-shot approach (top row). Generated using our proposed JKO-Flow using five iterations (bottom row). Here, we use α = 1, 5, 10, 50. JKO-Flow returns consistent results *regardless of the value of* α.

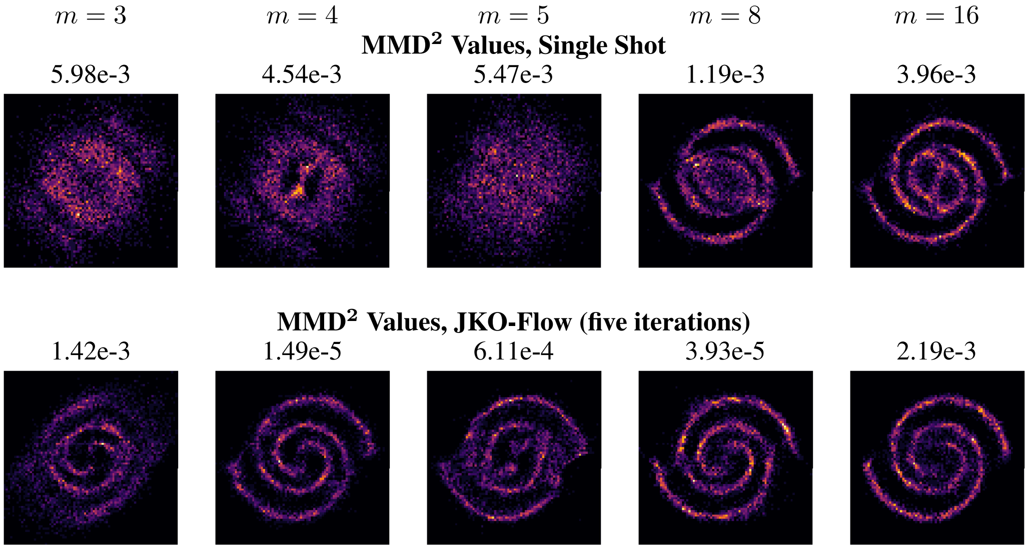

Supplementary Figure 7: 2 Spirals dataset: Generated samples of $\hat{\rho}_0$ using the standard single shot approach (top row). Generated samples using our proposed JKO-Flow using five iterations (bottom row). Here, we fix $\alpha = 50$ and vary the network width $m = 3, 4, 5, 8$, and 16. JKO-Flow performs competitively even with lower number of parameters.

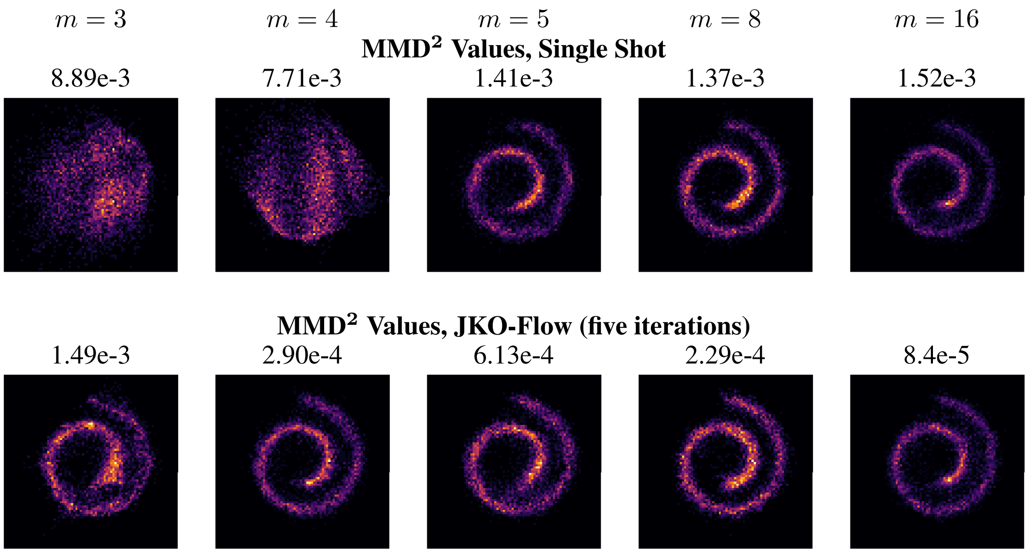

Supplementary Figure 8: Swiss Roll dataset: Generated samples of $\hat{\rho}_0$ using the standard single shot approach (top row). Generated samples using our proposed JKO-Flow using five iterations (bottom row). Here, we fix $\alpha = 5$ and vary the network width $m = 3, 4, 5, 8$, and 16. JKO-Flow performs competitively even with lower number of parameters.

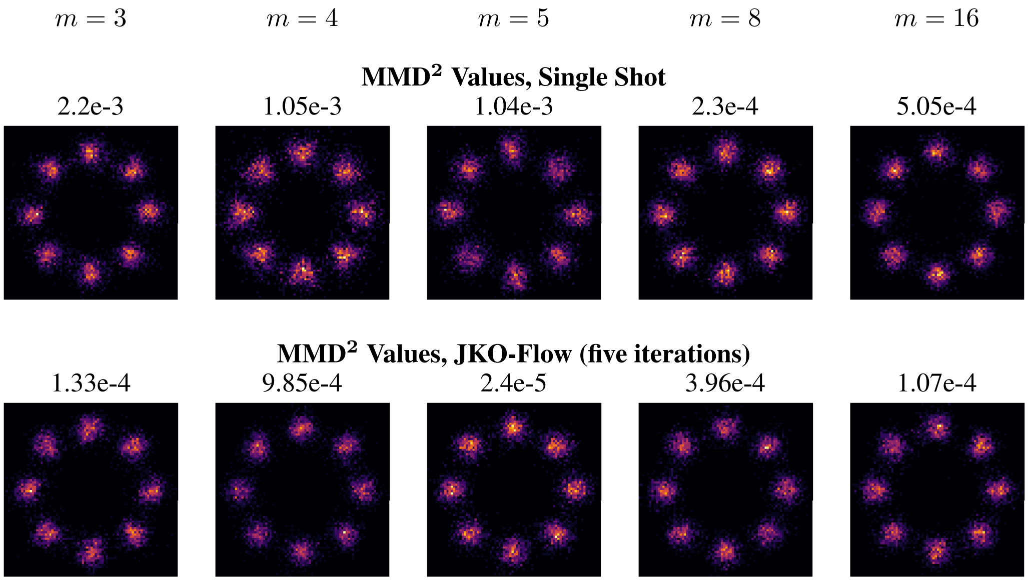

Supplementary Figure 9: 8 Gaussians dataset: Generated samples of $\hat{\rho}_0$ using the standard single shot approach (top row). Generated samples using our proposed JKO-Flow using five iterations (bottom row). Here, we fix $\alpha = 5$ and vary the network width $m = 3, 4, 5, 8$, and 16. JKO-Flow performs competitively even with lower number of parameters.

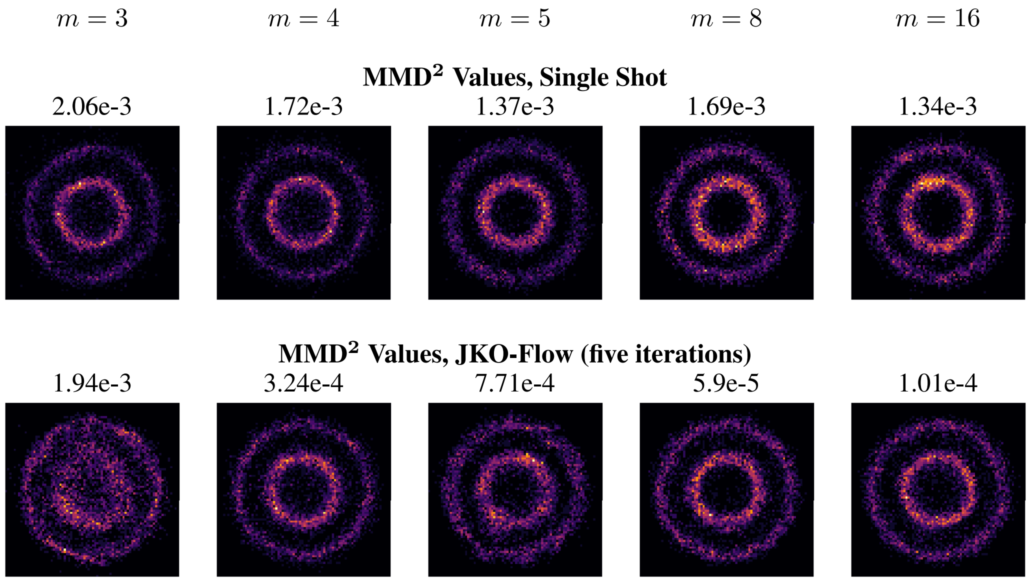

Supplementary Figure 10: Circles dataset: Generated samples of $\hat{\rho}_0$ using the standard single shot approach (top row). Generated samples using our proposed JKO-Flow using five iterations (bottom row). Here, we fix $\alpha = 5$ and vary the network width $m = 3, 4, 5, 8$, and 16. JKO-Flow performs competitively even with lower number of parameters.

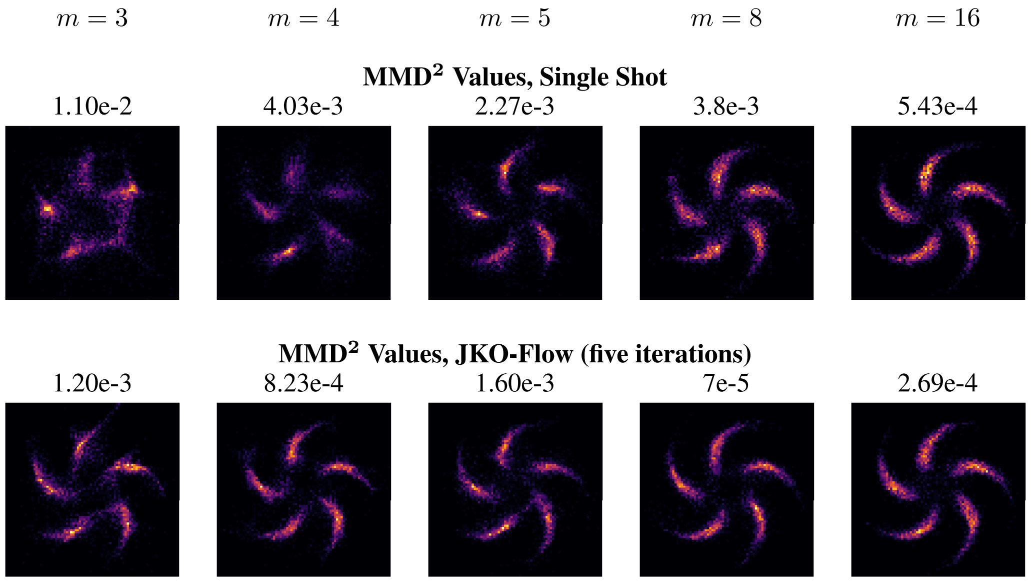

Supplementary Figure 11: Pinwheel dataset: Generated samples of $\hat{\rho}_0$ using the standard single shot approach (top row). Generated samples using our proposed JKO-Flow using five iterations (bottom row). Here, we fix $\alpha = 5$ and vary the network width $m = 3, 4, 5, 8$, and 16. JKO-Flow performs competitively even with lower number of parameters.

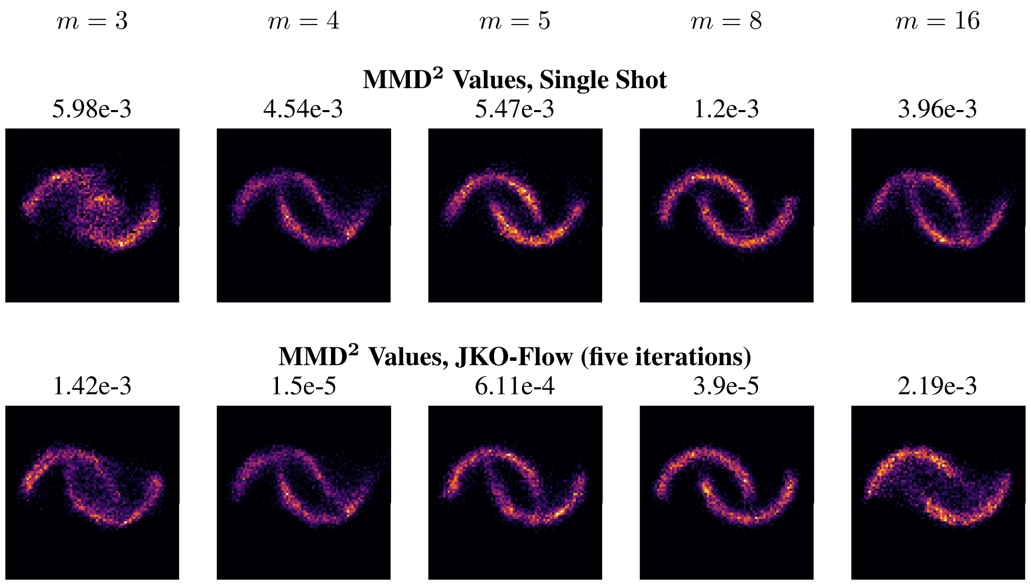

Supplementary Figure 12: Moons dataset: Generated samples of $\hat{\rho}_0$ using the standard single shot approach (top row). Generated samples using our proposed JKO-Flow using five iterations (bottom row). Here, we fix $\alpha = 5$ and vary the network width $m = 3, 4, 5, 8,$ and 16. JKO-Flow performs competitively even with lower number of parameters.

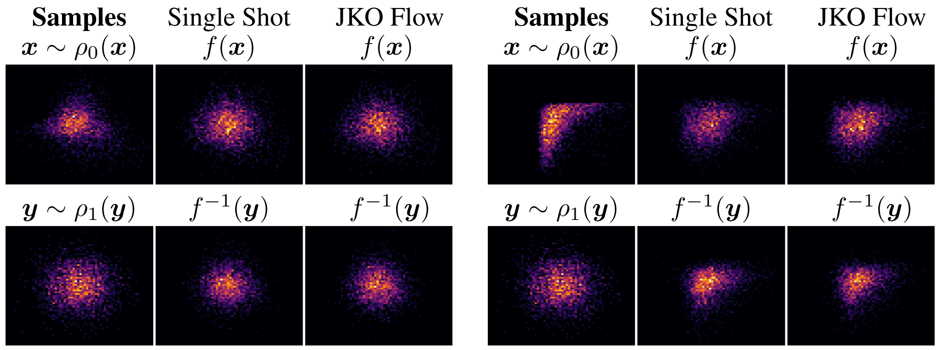

(a) MINIBOONE dimension 16 vs 17

(b) MINIBOONE dimension 28 vs 29

Supplementary Figure 13: Generated samples for the 43-dimensional MINIBOONE dataset using the single shot approach and JKO-Flow with 10 iterations for $\alpha = 1$. To visualize the dataset, we show 2-dimensional slices. We show the forward flow $f(x)$ where $x \sim \rho_0$ and the genereated samples $f^{-1}(y)$ where $y \sim \rho_1$.

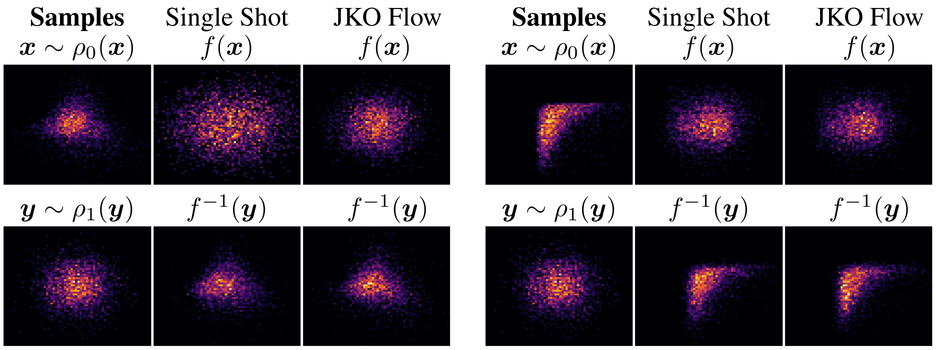

(a) MINIBOONE dimension 16 vs 17

(b) MINIBOONE dimension 28 vs 29

Supplementary Figure 14: Generated samples for the 43-dimensional MINIBOONE dataset using the single shot approach and JKO-Flow with 10 iterations for $\alpha = 10$. To visualize the dataset, we show 2-dimensional slices. We show the forward flow $f(x)$ where $x \sim \rho_0$ and the genereated samples $f^{-1}(y)$ where $y \sim \rho_1$.

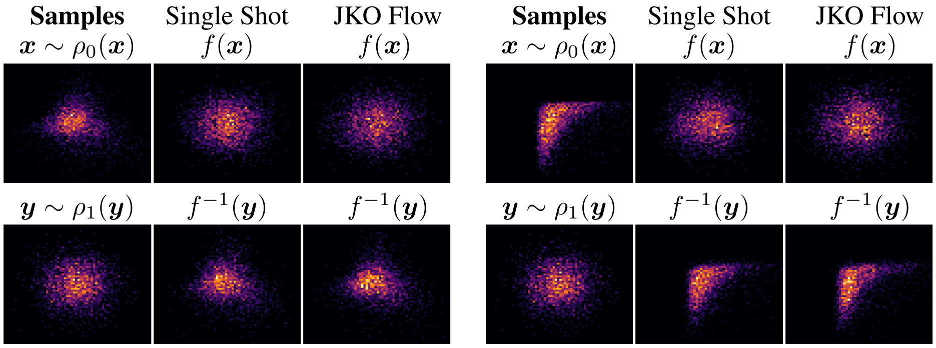

(a) MINIBOONE dimension 16 vs 17

(b) MINIBOONE dimension 28 vs 29

Supplementary Figure 15: Generated samples for the 43-dimensional MINIBOONE dataset using the single shot approach and JKO-Flow with 10 iterations for $\alpha = 50$. To visualize the dataset, we show 2-dimensional slices. We show the forward flow $f(x)$ where $x \sim \rho_0$ and the genereated samples $f^{-1}(y)$ where $y \sim \rho_1$.
